# A Unique Presentation of Occult Primary Breast Cancer with a Review of the Literature

**DOI:** 10.1155/2015/102963

**Published:** 2015-03-18

**Authors:** Inaya Ahmed, Kavita Dharmarajan, Amy Tiersten, Ira Bleiweiss, Hank Schmidt, Sheryl Green, Richard L. Bakst

**Affiliations:** ^1^Rutgers Robert Wood Johnson Medical School, New Brunswick, NJ 08901, USA; ^2^Department of Radiation Oncology, Icahn School of Medicine at Mount Sinai Hospital, New York, NY 10029, USA; ^3^Department of Medical Oncology, Icahn School of Medicine at Mount Sinai Hospital, New York, NY 10029, USA; ^4^Department of Pathology, Icahn School of Medicine at Mount Sinai Hospital, New York, NY 10029, USA; ^5^Department of Surgery, Icahn School of Medicine at Mount Sinai Hospital, New York, NY 10029, USA

## Abstract

We are reporting a case of a 34-year-old woman with occult primary breast cancer discovered after initially presenting with neurological symptoms. She was successfully treated with neoadjuvant chemotherapy followed by definitive axillary lymph node dissection and ipsilateral whole breast radiotherapy. The case presented is unique due to the rarity of occult primary breast cancer, especially in light of her initial confounding neurological signs and symptoms, which highlights the importance of careful staging.

## 1. Introduction

Occult primary breast cancer is defined as histologically proven breast cancer discovered outside the breast in the absence of a primary breast tumor. Occult primary breast cancer was first described in 1907 by Halsted. The incidence of occult primary breast cancer is 0.3–1.0% of all newly diagnosed breast cancers, and due to its rarity, its natural history has not yet been clearly documented [1]. Traditionally occult primary breast cancers were treated with mastectomy and axillary lymph node dissection; however, with the advent of breast MRI and improvement in radiation techniques, the management of this rare entity is changing. Here, we report a case of occult primary breast cancer with a unique initial presentation, and we review the currently available literature on diagnosis, treatment, and prognosis of this rare disease.

## 2. A Case Report

A 34-year-old woman with a history of headaches, seizure activity, and recently diagnosed breast cancer was initially referred to the Department of Radiation Oncology for irradiation of lesions in the brain concerning for metastases.

**Figure 1:**
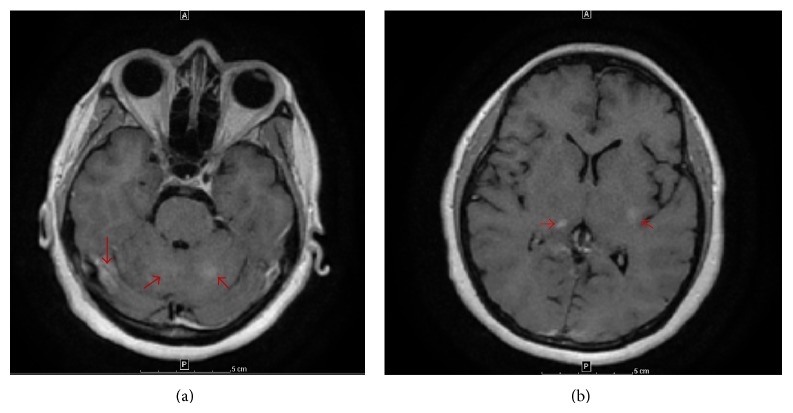
MRI brain: axial T1 images with contrast (a and b) demonstrating nodular enhancement (red arrows) in multiple areas of the brain.

One year prior to presenting to our clinic, she developed headaches and altered mental status. Magnetic resonance imaging (MRI) of the brain was suggestive of leptomeningeal enhancement with multiple nodular areas throughout the brain and brainstem (Figure 1). Further workup, including lumbar puncture, was nondiagnostic. While her initial symptoms spontaneously resolved, a similar episode occurred 6 months later. At this time she developed a large, palpable, right axillary node.

**Figure 2:**
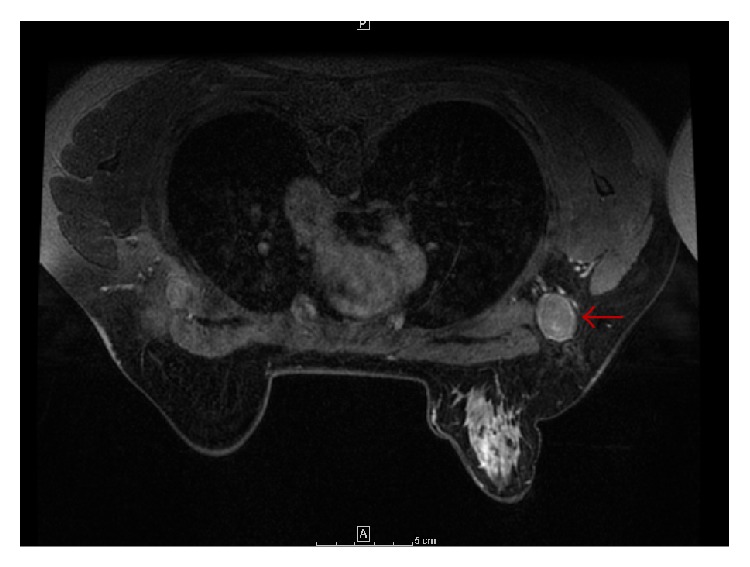
MRI breast: axial T1 image with contrast demonstrating normal breast tissue with right axillary lymphadenopathy (red arrow).

Diagnostic workup including an ultrasound imaging of the right axilla exhibited 2 large, adjacent, heterogeneous lymph nodes with loss of the fatty hila. Bilateral mammogram was suspicious for malignancy in the right breast, and follow-up MRI confirmed right axillary lymphadenopathy and also demonstrated extensive patchy enhancement in the right breast, involving the medial breast as well as the upper outer quadrant (Figure 2).

Ultrasound-guided biopsy of a right axillary lymph node revealed invasive ductal carcinoma. Immunohistochemistry was positive for HER2 and negative for estrogen and progesterone receptors. Stereotactic biopsy of the breast resulted in benign findings, demonstrating fibroadenoma, secretory changes, and stromal fibrosis with calcifications. Two subsequent MRI-guided biopsies were consistent with benign tissue. Staging positron emission tomography (PET) scan confirmed disease in the right axilla, with no FDG avidity in any other area, including the right breast and the areas corresponding with the brain lesions seen previously on MRI.

Repeat brain MRI exhibited progression of prior findings. In light of her new cancer diagnosis, it was felt that her MRI findings represented leptomeningeal carcinomatosis. She was therefore referred to the Department of Radiation Oncology for whole brain radiotherapy. However, scrutiny of her MRI and review of her prior imaging with both neurology and neuroradiology lead to a favored diagnosis of a primary granulomatous, rather than a metastatic, process in the brain. No biopsies were performed at the time.

In the absence of metastatic disease, the patient initiated definitive treatment consisting of neoadjuvant chemotherapy, consisting of four cycles of docetaxel, trastuzumab, and pertuzumab, followed by three cycles of 5-fluorouracil, epirubicin, and cyclophosphamide, which she tolerated well with no major toxicities.

**Figure 3:**
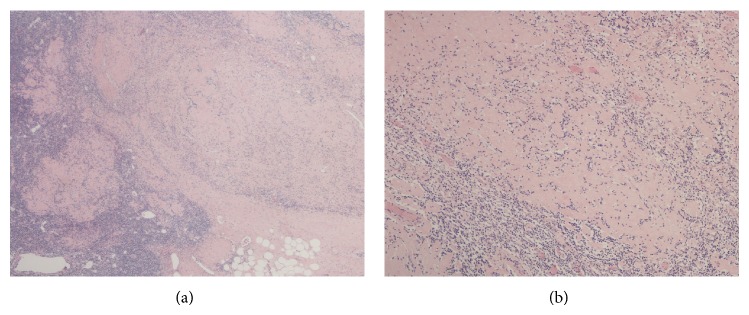
Axillary nodal pathology. Hematoxylin and eosin stained tissue slides from axillary lymph node dissection following chemotherapy. Low (a) and high power (b) from her lymph node showing large geographic areas of fibrosis where tumor was before chemotherapy.

**Figure 4:**
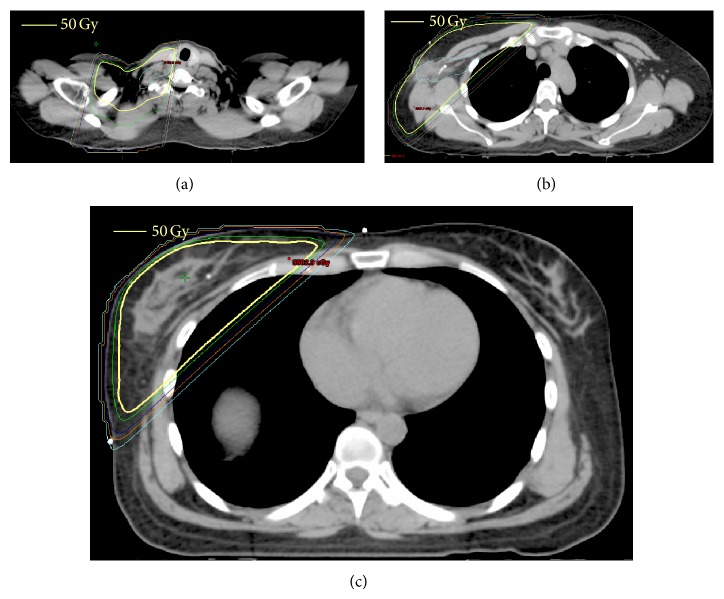
3D conformal radiotherapy plan for our patient: axial dose distribution to the supraclavicular fossa (a), axilla (b), and breast (c).

In lieu of undergoing mastectomy, the patient elected to undergo breast conserving therapy (BCT), which consists of axillary lymph node dissection and whole breast radiotherapy. All of the 22 lymph nodes dissected were negative for malignancy, though 2 nodes exhibited chemotherapy-related changes (Figure 3). After about 1.5 months of recovery, the patient initiated radiotherapy. She underwent external beam radiotherapy to 50 Gy in 25 daily fractions to the right breast, axilla, and supraclavicular fossa (Figure 4).

Three months after completion of radiotherapy, she was not exhibiting any side effects or residual toxicity and continues to be free of headaches or neurological symptoms.

## 3. Discussion

Rarely, a breast cancer patient has histologically proven breast cancer found outside the breast without a detectable primary breast tumor. Almost always the site of detected disease is an axillary lymph node. The incidence of axillary breast cancer with occult primary is 0.3–1.0%, with a peak incidence at the age of 55 [1, 2]. Historically, a relatively large proportion of patients (20–30%) with occult primary breast cancer have reported a family history of breast cancer, although this was not the case for our patient [8].

Diagnostic workup for an axillary metastasis must first rule out other primary sites of disease. This may involve history and physical (including full skin exam for melanoma), chest X-ray, mammography, PET scan, hepatic imaging, and serum studies [9]. Dedicated imaging of the breast for occult primary breast cancer includes breast ultrasound and MRI. MRI's high sensitivity for breast tumors may optimize diagnostic accuracy and, in turn, disease management [10]. If a primary site is identified, the patient should undergo appropriate surgical management with a lumpectomy or mastectomy with lymph node evaluation.

Occult primary breast cancer has traditionally been treated with ipsilateral mastectomy and axillary lymph node dissection (ALND). However, practice patterns have steadily transformed over the last two to three decades with a push toward BCT [3]. In the case of occult primary breast cancer, BCT entails ipsilateral ALND and radiotherapy to the whole breast. Whole breast radiotherapy and mastectomy have been associated with statistically equivalent outcomes in several studies, with a 10-year overall survival of 65% [1, 3–5]. In comparison, a 2010 SEER database analysis reported that patients treated with ALND only or observation without intervention exhibited diminished survival rates of 58.5% (*P* = 0.02) and 47.5% (*P* = 0.04), respectively [3]. Furthermore, failure to provide any intervention to the ipsilateral breast is unacceptable, as locoregional failure in those cases may exceed 80% [6].

Disease recurrence patterns are also reported to be similar among patients treated with either mastectomy or BCT. In a 2012 retrospective analysis of 95 patients treated with either mastectomy (with ALND), BCT, or ALND only, patients treated with mastectomy or BCT exhibited a 3-year local recurrence-free survival (LRFS) of 88.9–90.0% and overall recurrence-free survival (RFS) of 71.9–81.6%, with no difference between the two cohorts (*P* = 0.71, 0.94) [4]. Patients treated solely with ALND demonstrated significantly diminished 3-year LRFS (70.2%) and overall RFS (52.1%). These results are comparable to those of prior smaller, retrospective studies, reporting similar recurrence rates among patients undergoing mastectomy or BCT and poor outcomes in patients without any treatment to the ipsilateral breast [1, 7, 11]. Accordingly, local therapy to the ipsilateral breast, either mastectomy or BCT, plays a crucial role in the treatment of occult primary breast cancer. These studies are summarized in Table 1.

From the available retrospective literature, data regarding disease prognosticators remains relatively elusive. Many of the known prognositc factors in primary breast cancer have been investigated, including age, tumor grade, hormone receptor status, HER2/neu status, and nodal stage [1, 3–5]. To date, only nodal status has been consistently associated with survival outcomes in occult primary breast cancer. In the 2010 SEER analysis, Walker et al. found that patients with ≥10 positive lymph nodes had worse overall survival (*P* < 0.01) and cause-specific survival (*P* = 0.02) than those with <10 nodes positive [3]. Smaller retrospective studies have shown a significant improvement in survival when patients have 1–3 versus ≥4 positive lymph nodes [1, 4, 5]. Although extent of nodal disease clearly influences prognosis, the importance of specific nodal sites (i.e., axillary versus infraclavicular) remains unclear.

Due to the rarity of occult primary breast cancer, much about this disease has yet to be fully defined. With the incidence of this presentation too infrequent for prospective study, therapeutic decisions are synthesized utilizing the available retrospective data. In patients with occult primary breast cancer, thorough diagnostic evaluation is crucial in ruling out other primary malignancies, and breast MRI is standard to confirm the absence of a primary breast tumor. In terms of definitive therapy for occult primary breast cancer, BCT or mastectomy with ALND provides equivalent survival and recurrence outcomes, and the practice of BCT is steadily increasing. Furthermore, nodal status serves as the main prognostic factor in these cases. With definitive treatment with BCT and only 2 positive lymph nodes, we expect an excellent prognosis for our patient. Lastly, this case reminds us that careful staging of any patient is critical. If not for a thoughtful diagnostic process and careful interdisciplinary approach to patient management decisions at our institution, our patient may have received unnecessary therapy for presumed brain metastases and have not received definitive treatment.

## Figures and Tables

**Table 1:** Summary of literature reviewed on occult primary breast cancer.

	Median F/U (yr)	Patients	Overall survival		Recurrence-free survival		Locoregional recurrence	Distant recurrence
			5 yr OS	10 yr OS	5 yr RFS	10 yr RFS		
Vlastos et al. (2001) [1]	7.0	BCT: 32	79%	64%	72%	63%	13%	22%
		Mast: 13	75%	66%	67%	56%	15%	31%

Galimberti et al. (2004) [2]	3.4	50	—	—	—	—	4%	12%

Walker et al. (2010) [3]	4.0	BCT: 202		67.1%	—	—	—	—
		Mast: 268	—	63.5%	—	—	—	—
		ALND: 126	—	58.5%^*^	—	—	—	—
		Obs: 94	—	47.5%^*^	—	—	—	—

He et al. (2012) [4]	3.2	BCT: 13	—	—	—	—	8%	15%
		Mast: 64	—	—	—	—	11%	6%
		ALND: 18	—	—	—	—	28%	6%

Campana et al. (1989) [5]	9.0	31	76%	71%	86%	75%	25%	29%

Foroudi and Tiver (2000) [6]	9.2	BCT: 12	—	—	—	—	25%	0%
		Mast: 2	—	—	—	—	0%	0%
		Obs: 6	—	—	—	—	83%	—

Barton et al. (2011) [7]	5.7	BCT: 35	84%	—	64%	—	14%	—
		No RT: 13 (nodal and/or partial breast resection)	85%	—	34%^*^	—	85%	—

(i) ^*^Statistically significant value.

(ii) BCT: breast conserving therapy, RT: radiotherapy, Mast: mastectomy, ALND: axillary lymph node dissection, and Obs: observation.

(iii) OS: overall survival, RFS: recurrence-free survival, F/U: follow-up period, and mo: month.

(iv) —: not reported.

## References

[1] Vlastos G., Jean M. E., Mirza A. N. (2001). Feasibility of breast preservation in the treatment of occult primary carcinoma presenting with axillary metastases. *Annals of Surgical Oncology*.

[2] Galimberti V., Bassani G., Monti S. (2004). Clinical experience with axillary presentation breast cancer. *Breast Cancer Research and Treatment*.

[3] Walker G. V., Smith G. L., Perkins G. H. (2010). Population-based analysis of occult primary breast cancer with axillary lymph node metastasis. *Cancer*.

[4] He M., Tang L. C., Yu K. D. (2012). Treatment outcomes and unfavorable prognostic factors in patients with occult breast cancer. *European Journal of Surgical Oncology*.

[5] Campana F., Fourquet A., Ashby M. A. (1989). Presentation of axillary lymphadenopathy without detectable breast primary (T_0_ N_ib_ breast cancer): experience at Institut Curie. *Radiotherapy & Oncology*.

[6] Foroudi F., Tiver K. W. (2000). Occult breast carcinoma presenting as axillary metastases. *International Journal of Radiation Oncology Biology Physics*.

[7] Barton S. R., Smith I. E., Kirby A. M., Ashley S., Walsh G., Parton M. (2011). The role of ipsilateral breast radiotherapy in management of occult primary breast cancer presenting as axillary lymphadenopathy. *European Journal of Cancer*.

[8] Harrington S. W. (1946). Survival rates of radical mastectomy for unilateral and bilateral carcinoma of the breast. *Surgery*.

[9] Halperin E. C., Perez C. A., Brady L. W. (2008). *Perez and Bradys Principles and Practice of Radiation Oncology*.

[10] National Comprehensive Cancer Network (2014). *Breast Cancer (Version 3.2014)*.

[11] Varadarajan R., Edge S. B., Yu J., Watroba N., Janarthanan B. R. (2007). Prognosis of occult breast carcinoma presenting as isolated axillary nodal metastasis. *Oncology*.

